# Revisit of Polyomavirus Nephropathy Grading in Renal Allograft Recipients According to the Banff 2019 Working Group Classification: A Study From a Large Transplant Center in South India

**DOI:** 10.7759/cureus.22377

**Published:** 2022-02-19

**Authors:** Kalaivani S Subramanian, Bheemanathi Hanuman Srinivas, Rajesh Nachiappa Ganesh, Debasis Gochhait, Priyamvada PS, Sreejith Parameswaran, Sathish Haridasan

**Affiliations:** 1 Department of Pathology, Sri Venkateshwaraa Medical College Hospital and Research Centre, Puducherry, IND; 2 Department of Pathology, Jawaharlal Institute of Postgraduate Medical Education and Research, Puducherry, IND; 3 Department of Nephrology, Jawaharlal Institute of Postgraduate Medical Education and Research, Puducherry, IND

**Keywords:** polyomavirus nephropathy, banff 2019 classification, outcome, clinical presentation, histopathology, bk virus

## Abstract

Background

In renal transplant patients, the biopsy-proven incidence of polyomavirus nephropathy (PVN) is approximately 5%. There is no consensus in the morphologic classification of definitive PVN, which is attempted in the Banff 2019 Working Group classification, which groups histologic changes, reflects clinical presentation, and facilitates comparative outcome analyses. This study aims to analyze the clinical and histopathological findings and outcomes among the three classes in the recent classification.

Materials and methods

The study was conducted in the department of pathology and nephrology over a period of six years. All cases diagnosed as PVN on renal allograft biopsies were included. The clinical and biochemical findings were obtained from hospital records. Histopathology slides were reviewed and classified according to Banff 2019 criteria and were analyzed with clinical, laboratory, histopathological parameters along with the clinical outcome.

Results

Out of 205 renal transplants performed during the study period, 14 patients (6.8%) were diagnosed with PVN. The mean age of diagnosis was 38 years, with a Male: Female ratio of 1.8:1. The median period of diagnosis of the viral infection after transplant was 10 months. Histomorphology grading according to Banff 2019 revealed four cases (28.5%) in PVN class 1, eight cases (57.2%) in PVN class 2, and two cases (14.3%) in PVN class 3. Cases in PVN class 1 presented early. PVN class 1 was associated with a single type of inclusion, and multiple type inclusions were observed in higher classes. Associated diseases were thrombotic microangiopathy (TMA), borderline cellular rejection, antibody-mediated rejection (ABMR), and concomitant infections. PVN class 1 had a better outcome compared to PVN class 2 and class 3.

Conclusion

PVN1 was observed to have better clinical presentation and outcomes than PVN2 and 3; however, this could not be statistically concluded due to the low sample size and other associated diseases.

## Introduction

Polyomavirus nephropathy (PVN) is a common viral infection in renal transplant recipients [1-2]. Polyomavirus infection is usually caused by the BK virus (BKV), which occurs in childhood and becomes latent in urinary tract epithelial cells. It is reactivated in immunosuppressed individuals [3-4]. The biopsy-proven incidence of PVN is approximately 5-6% [5-6]. Previous classifications schemas based on the histological changes of PVN neither have broad acceptance nor clinical relevance [7-8]. The Banff community created a working group with the goal of developing a clinically relevant morphological classification of PVN. The Banff working group classification (2019) is considered more comprehensive, correlates well with the clinical presentation, and facilitates the comparative outcome analyses of PVN [9]. Thus this study aimed at the revision of the histomorphological grading of PVN cases according to the Banff 2019 classification and the analysis of the clinical and histomorphological features and clinical outcomes in various classes of polyomavirus nephropathy.

This article was previously presented as a poster and oral paper at the Annual Conference and CME of the Indian Society of Renal and Transplantation Pathology (ISRTPCON) in October 2019.

## Materials and methods

This is a retrospective study conducted in the department of pathology and nephrology. All cases diagnosed as PVN on renal allograft biopsies for a period of six years were included. Clinical and biochemical data were collected from hospital records using a predefined proforma. Histopathology slides (H&E, special histochemical stains, SV 40, C4d) were reviewed and classified according to the Banff 2019 criteria by two pathologists who were blinded to the clinical data.

The parameters analyzed were age, sex, clinical presentation, time of biopsy from transplantation, serum creatinine levels, BKV DNA levels in urine and serum, associated diseases, and clinical outcomes. The histopathological findings, including the glomerular/vessel changes, acute tubular necrosis, inflammation, type of inclusions, pvl (polyomavirus load), and ci (fibrosis) score were evaluated and scored according to Banff 2019, and these were correlated with the three classes of the PVN classification according to the Banff 2019 criteria

The intrarenal pvl was semi-quantitatively scored on the basis of the overall percentage of tubules with morphologic evidence of polyomavirus replication. According to this definition, a tubule with intranuclear viral inclusion bodies and/or a diagnostic immunohistochemical (IHC0 staining reaction for the SV40-T antigen in one or more tubular epithelial cells per tubular cross-section was considered “one positive tubule. The percentage positive was on the basis of an evaluation of all available cores in the biopsy. Three levels of pvl were defined: pvl 1: <1%; pvl 2: 1%-10%; pvl 3: >10% positive tubules/ducts. The Banff interstitial fibrosis score was grouped in a binary fashion (i.e., ci2 >25% or ci1 <25%). The classification based on the pvl score and ci score divided PVN into three classes PVN class 1: pvl 1, ci 1; PVN; Class 2: pvl 1, ci2, or pvl 2, any ci score or pvl 3, ci1 and PVN class 3: pvl 3, ci2

The demographic details and distribution of various parameters in the three PVN classes were expressed as frequency, and the parameters were correlated with the three PVN classes manually.

## Results

**Figure 1:**
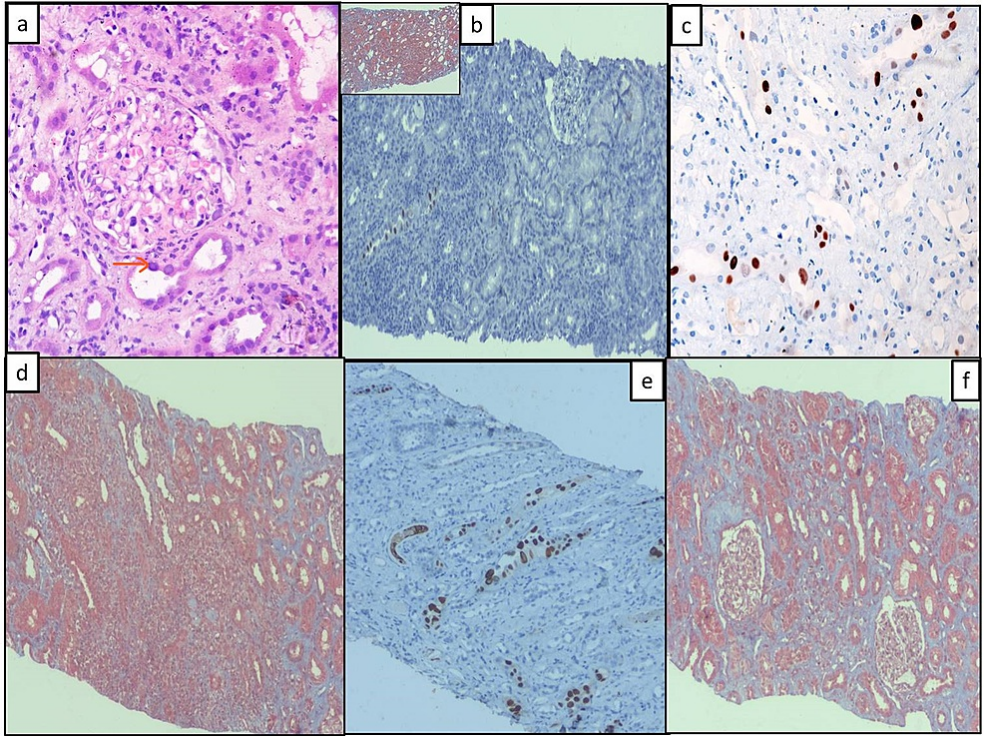
Histopathological and immunohistochemical features of polyomavirus nephropathy Kidney biopsy (a&b: PVN1): a) shows enlarged tubular epithelial cells with perinuclear halo and smudged nuclear chromatin (H&EX200); b) IHC with SV40 shows occasional tubule showing positivity (DABX100); inset reveals no fibrosis on Mason's trichrome stain; (c&d: PVN2): c) IHC with SV40 shows few tubules showing positivity (DABX200); d) focally increased fibrosis with moderate to dense interstitial inflammatory infiltrate (MTX100): (e&f: PVN3); e) IHC with SV40 reveals much tubular positivity (DABX200); f) shows marked interstitial widening with large areas of fibrosis (MTX100) IHC: immunohistochemistry; H&E: hematoxylin and eosin

Out of 205 renal transplants performed during the study period, 14 patients (6.8%) were diagnosed as PVN based on renal histology as well as SV40 immunohistochemistry. The mean age of diagnosis is 38 years with a Male: Female ratio of 1.8:1. The median period of diagnosis of viral infection after transplant was 10 months (Range 3 months - 4.4 years). Serum creatinine at the time of biopsy ranged from 1.4 mg to 4.4 mg/dl. The BKV viral load in urine and serum was available for 12 patients; viruria was detectable and quantifiable in all cases; however, viremia was detected only in 8/12 cases. Histomorphology graded according to Banff 2019 revealed four cases (28.5%) in PVN class 1 (Figures 1a-1b), eight cases (57.2%) in PVN class 2 (Figure 1c-1d), and two cases (14.3%) in PVN class 3 (Figure 1e-1f).

The detailed clinical and laboratory parameters in the three PVN classes are tabulated in Table 1. PVN class 1 cases presented early with high serum creatinine. The histopathological parameters are also tabulated in Table 1. Acute tubular necrosis and interstitial inflammation increased with the increase in PVN classes. PVN class I was associated with a single type of inclusion, commonly type 1 and multiple types of inclusions were observed in higher classes. Type 1 inclusion was seen in all cases followed by type II and types III and IV inclusions. Associated diseases were TMA, borderline cellular rejection, ABMR, and infections as tabulated in Table 1. PVN class 1 had a better outcome as compared to PVN class 2 and class 3.

**Table 1:** Clinical, laboratory, histopathological parameters and associated diseases in PVN classes CNI - calcineurin inhibitors, ATN - acute tubular necrosis, pvl - polyomavirus load, ci - fibrosis, FSGS - focal segmental glomerulosclerosis, ACR - acute cellular rejection, ABMR - acute antibody-mediated rejection, TMA - thrombotic microangiopathy

CLINICAL AND LABORATORY PARAMETERS			
PARAMETERS	PVN CLASS 1(4)	PVN CLASS 2(8)	PVN CLASS 3(2)
Age (yrs) (mean)	19-51 (34)	15-40 (30)	47-53 (50)
Serum creatinine at index biopsy mg/dl (mean)	1.8-6.5 (4.1)	1.4-2.2 (1.8)	2 (2)
Time from transplant to biopsy (months) (mean)	3-10 (5.8)	4- 52.8 (20.8)	9-14 (11.5)
Clinical diagnosis (n)	PVN (1) Rejection/CNI (2) Graft dysfunction (1)	Rejection/PVN (5) Rejection/CNI toxicity(3)	Rejection/PVN (1) Rejection(1)
BKV DNA in serum and urine (n-12)	Urine -3 Serum-2	Urine -8 Serum -5	Urine and serum -1
HISTOPATHOLOGICAL PARAMETERS			
Glomeruli/Vessels (n)	ABMR (1)	TMA (1) FSGS (1)	ABMR(1)
ATN (%) (mean)	10-30 (20)	5-30 (15)	20-30(25)
Inflammation (n)	i1 (1) i2 (3)	i1 (2) i2 (5) i3 (1)	i2(1) i3(1)
pvI, ci (n)	pvl1, ci0 (2) pvl1,ci1(2)	pvl2, ci0(3), pvl2, ci1 (2), pvl2, ci2 (2), pvl3, ci1(1)	pvl3, ci2(2)
Types of inclusion (n)	Type I (3), Multiple types (1)	Type I (4), Multiple (4)	Multiple (2)
ASSOCIATED DISEASES			
Pre-biopsy (n)	ACR & ABMR (1)	ABMR (1)	No significant graft disease
Present biopsy (n)	ABMR (1), Borderline cellular rejection (1), Acute pyelonephritis (2)	TMA (1), Borderline cellular (1)	ABMR (1), Borderline cellular rejection (1)
Follow-up biopsy (n)	-	Chronic ABMR(1)	Cutaneous fungal infection(1)

There were no standard treatment protocols followed; treatment was individualized based on the patient’s comorbidities and other disease associations. The usual treatment received is a reduction of the Tacrolimus dose to the target of 4-5 ng/ml. For some patients, Mycophenolate mofetil (MMF) is either reduced to 50% or completely stopped. Leflunomide and ciprofloxacin were added in a few, and some received intravenous immunoglobulins (IVIg) also.

On follow-up, three of the four cases of PVN 1 who had a follow-up showed improved clinical outcomes with a reduction in creatinine level (serum creatinine at index biopsy - 1.8- 6.5 mg/dl) to normal creatinine levels and nondeductible viral load in serum and urine. One patient had a repeat biopsy, which showed clearance of PVN.

Six of PVN 2 had a follow-up, one patient expired due to the development of the extrarenal post-transplant lymphoproliferative disorder (PTLD) - diffuse large cell lymphoma involving the liver and spleen (serum creatinine was normal with no viremia/viruria, no PVN on repeat renal biopsy), one patient developed graft failure (serum creatinine rose to 6.8 mg/dl; the patient also had associated TMA), and four had persistent dysfunction (serum creatinine 1.4 to 2.06 mg/dl), three of which had persistence of PVN on repeat biopsy.

Both cases of PVN3 had a poor outcome, one expired and the other developed persistent renal dysfunction (serum creatinine increased from the time of index biopsy).

## Discussion

The goal of the new Banff criteria is “developing a clinically relevant morphologic classification for PVN” that is based on the polyomavirus load (pvl) and interstitial fibrosis (ci) [9]. We tried to reclassify polyomavirus nephropathy based on the new Banff criteria and tried to observe the various clinical, laboratory, and histopathological parameters in the three groups. The incidence of PVN in our study is 6.8%, which is slightly less than the study by Sachdeva MS et al. (9.3%) [10] but similar to a previous study by Vinitha et al, (4%) [11]. There are only very few studies available on PVN using the current Banff criteria [9,12]. In the study cohort by Nickeleit V et al., PVN was diagnosed in the index biopsy between four and 582 weeks post-transplantation (median of 28 weeks) [9]. In our study, the median period of diagnosis of viral infection after transplant is 10 months (three months - 4.4 years). The mean period of diagnosis of viral infection after transplant was 12.4 months (seven days to 3.5 years) for PVN by Vinitha et al. [11], and it is 10.6 weeks to 140 weeks in the recent validation study by Nickeleit et al. In addition, the time of diagnosis of PVN cases strongly correlated with PVN classes, which were 16.9 weeks for PVN 1, 24.2 weeks for PVN2, and 58.7 weeks for PVN 3 [12].

In the study cohort by Nickeleit V et al. [9], 25% of patients (44 of 178) were categorized as PVN class 1, 63% (112 of 178) were categorized as class 2, and 12% (22 of 178) were categorized as class 3, which is comparable with our study, which showed four cases (28.5%) in PVN class 1, eight cases (57.2%) in PVN class 2, and two cases (14.3%) in PVN class 3.

In the original study cohort by Nickeleit V et al. [9], PVN class 1 was diagnosed early: 18 weeks (median) after grafting in patients with a modest rise in serum creatinine levels. In comparison, PVN classes 2 and 3 were diagnosed later with medians of 30 and 54 weeks after transplantation, respectively; with more pronounced increases in serum creatinine levels compared with baseline. Similar findings were observed in the recent validation study by Nickeleit et al., where PVN 1 was detected earlier than PVN 2/3 [12]. In our study also, PVN class 1 presented early than PVN classes 2 and 3 as observed in the cohort and validation study but unlike these studies, the rise in serum creatinine was higher in PVN class 1 as compared to PVN classes 2 and 3. This could be because all the cases of PVN class 1 with high serum creatinine were associated with comorbidities like either pyelonephritis or rejection.

In the study cohort by Nickeleit V et al., intranuclear viral inclusions were absent in 48%, however, the diagnosis of PVN was confirmed with IHC. In our study, IHC was done only for 49 cases based on clinical and morphological suspicion, out of which 14 cases turned out to be positive and all cases showed the presence of viral inclusions, of which type I was the common type of inclusion identified. It is also observed in our study, and we would like to highlight that as the viral load increases, multiple types of inclusions were identified. And the amount of inflammation also increased with higher PVN classes as observed by Nickeleit V et al. [9].

During follow-up in the study cohort by Nickeleit V et al., 16% of grafts failed in PVN class 1, 31% of grafts failed in PVN class2, and 50% of grafts failed in PVN class 3 [9]. In the validation study by Nickeleit et al., 5% of grafts failed in PVN class 1, 30% of grafts failed in PVN class 2, and 50% of grafts failed in PVN class 3 [12]. Though our study population was small and could not be statistically validated, our study also showed a similar pattern of outcomes, with PVN class 1 showing better outcomes than PVN classes 2 and 3, which were associated with persistent dysfunction, graft failure, and mortality.

In PVN1, three cases who had a follow-up had better outcomes; their serum creatinine returned to normal with no detectable BKV DNA in urine or serum. Six of the PVN 2 cases had a follow-up, and all had poor outcomes; one patient expired due to the development of extrarenal post-transplant lymphoproliferative disorder involving the liver and spleen, a biopsy from the kidney did not show viral inclusion or PTLD (serum creatinine was normal with no viremia/viruria), one patient developed graft failure (serum creatinine raised to 6.8 mg/dl; the patient also had associated TMA), and four had persistent dysfunction (serum creatinine 1.4 to 2.06 mg/dl, however, the creatinine levels decreased compared to the time of index biopsy), three of which had persistence of PVN on repeat biopsy. BKV DNA was on a rising trend in one patient and the others showed a reduction in viral load. Both cases of PVN3 had poor outcomes, one expired and the other developed persistent dysfunction (serum creatinine increased from the time of index biopsy, with detectable BKV DNA level on follow-up).

In our study, 50% of the cases had associated diseases like rejection, pyelonephritis, thrombotic microangiopathy (TMA), and cutaneous infections like phaeohyphomycosis, which could have also influenced the outcome. Three patients have been diagnosed as PVN with associated borderline cellular rejection in the biopsy (one in each PVN class). Though interstitial inflammation and tubulitis can be part of PVN-associated injury, borderline cellular rejection was suggested in these cases based on the presence of these features away from the areas of tubular injury due to viral inclusions. In spite of these disease associations in all PVN classes, PVN class 1 had a better outcome. Hence, the Banff 2019 PVN classification, despite other associations, may still play an important role in predicting outcomes.

Our study could not conclude anything statistically due to the less sample size. More studies with a larger sample size need to be conducted to further validate this classification to compare the outcome in all three PVN classes, including and excluding the associated complications

## Conclusions

Histomorphologically graded according to Banff 2019, 28.5% of cases were in PVN class 1, 57.2% cases in PVN class 2, and 14.3% of cases in PVN class 3. PVN class 1 cases present early. PVN class 1 was associated with a single type of inclusion, and multiple types of inclusions were observed in higher classes. The associated diseases were thrombotic microangiopathy (TMA), borderline cellular rejection, antibody-mediated rejection (ABMR), pyelonephritis, and other concomitant infections. PVN1 was observed to have a better clinical presentation, graft function, and outcome than PVN2 and 3.
